# An Efficient Parallel Algorithm for Multiple Sequence Similarities Calculation Using a Low Complexity Method

**DOI:** 10.1155/2014/563016

**Published:** 2014-07-22

**Authors:** Evandro A. Marucci, Geraldo F. D. Zafalon, Julio C. Momente, Leandro A. Neves, Carlo R. Valêncio, Alex R. Pinto, Adriano M. Cansian, Rogeria C. G. de Souza, Yang Shiyou, José M. Machado

**Affiliations:** ^1^Department of Computer Science and Statistics, Sao Paulo State University, Rua Cristóvão Colombo 2265, 15054-000 São José do Rio Preto, SP, Brazil; ^2^Department of Control Engineering and Automation, Federal University of Santa Catarina, Rua Pomerode 710, 89065-300 Blumenau, SC, Brazil; ^3^College of Electrical Engineering, Zhejiang University, Hangzhou 310027, China

## Abstract

With the advance of genomic researches, the number of sequences involved in comparative methods has grown immensely. Among them, there are methods for similarities calculation, which are used by many bioinformatics applications. Due the huge amount of data, the union of low complexity methods with the use of parallel computing is becoming desirable. The *k-mers* counting is a very efficient method with good biological results. In this work, the development of a parallel algorithm for multiple sequence similarities calculation using the *k-mers* counting method is proposed. Tests show that the algorithm presents a very good scalability and a nearly linear speedup. For 14 nodes was obtained 12x speedup. This algorithm can be used in the parallelization of some multiple sequence alignment tools, such as MAFFT and MUSCLE.

## 1. Introduction

The use of sequence comparison methods has been remarkably growing in recent years, in response to the data expansion of genomic research. Consequently, methods that reduce the execution time are fundamental to the progress of this area. Many efforts have been made concerning their optimization [1, 2].

With the increase in number of multiple sequences alignments problems, the development of methods which have a lower computational complexity also increases. Nevertheless, just the creation of low complexity methods was not enough to work with high volume of data [3, 4]. The interest in parallel computing has grown and, hence, several parallel methods were created and embedded in many sequence comparison tools [5].

In general, the sequence comparison starts with the similarity calculation between pairs of sequences. It occurs through methods which require or not a prior alignment, varying the algorithm complexity and the level of biological accuracy. The alignment free methods correspond to a class of low complexity methods, which have a low temporal and spatial complexity in relation to the methods which requires a prior alignment. Moreover, they also keep a high level of biological accuracy for divergent sequences. For this reason, they are becoming increasingly important in computational biology.

The similarity calculation methods shall be classified into two main categories: methods based on the counting of words and methods that do not involve such a counting. Among the methods based on the counting of words there is the* k-mers* counting method, which was proposed by Katoh et al. [6]. This method counts the number of* k-mers* (words of size* k*) shared by a pair of sequences, using it as an approximation of the similarity level. It also uses a different alphabet, built on the basis of statistical data, maximizing the biological accuracy in relation to the previously proposed methods.

The* k-mers* counting method was implemented in some important multiple sequences alignment tools, such as MAFFT [6, 7] and MUSCLE [1], and to the extent of our knowledge there is no other work in the literature that has developed and tested a parallel approach, specifically of this method. Once they are important tools, giving excellent results, in both biological accuracy and computational complexity [8], the development of a parallel algorithm for multiple sequence similarities calculation using the* k-mers* counting method is very useful. Although a parallel MUSCLE exists for shared memory systems [3], it does not include the parallelization of this stage. The parallel algorithm can be used in the development of any parallel tool that requires it in one of your stages. Tree construction or progressive multiple alignment tools, in general, are some examples.

This paper presents the* k-mers* counting method with the proposed similarity calculation parallel algorithm for multiple sequences through this method. The algorithm was developed for distributed memory parallel systems, using the library MPI [9].

## 2. Materials and Methods

### 2.1. Word Counting Methods

In general, word counting methods start with the mapping from sequences to vectors which store the length of each word. These words are subsequences of length *k*, also known as a *k*-tuple.

In order to understand the behavior of these methods, it is interesting to perform a review about some words statistical concepts. Then, consider a sequence *X*, of length *n*, defined as a segment of *n* symbols of a finite alphabet *A*, of length *r*.

A segment of *k* symbols, with *k* ≤ *n*, is a *k*-tuple. The set *W*_*k*_ consists of all possible *k*-tuples from the alphabet set *A* and has *N* elements:

(1) $$\begin{array}{c} W_{k}=\left\{w_{k,1},w_{k,2},\ldots ,w_{k,N}\right\},\\ N=r^{k}. \end{array}$$

Then, we count the number of *k*-tuples of *W*_*k*_ which appear in the sequence *X*. Computationally, this count is normally made moving a window of size *k* through the sequence, from the position 1 until the position *n* − *k* + 1. The vector *c*_*k*_^*X*^ is responsible for the storage of the number of occurrences of *k*-tuples in the sequence *X*:

(2) $$\begin{array}{c} c_{k}^{X}=\left(c_{k,1}^{X},c_{k,2}^{X},\ldots ,c_{k,N}^{X}\right). \end{array}$$

A frequency vector *f*_*k*_^*X*^ can then be gotten from the relative quantity of each *k*-tuple:

(3) $$\begin{array}{c} f_{k}^{X}=\frac{c_{k}^{X}}{\sum_{j=1}^{N}c_{k,j}^{X}}\equiv f_{k,i}^{X}=\frac{c_{k,i}^{X}}{n-k+1}. \end{array}$$

As an example of the use of these structures, imagine a DNA sequence, where *A* = {*A*, *T*, *C*, *G*} and *r* = 4. For *k* = 3, *ATC* and *AAA* are *k*-tuple belonging to the set *W*_3_. For the sequence *X* = *ATATAC*, where *n* = 6, the counting and frequency vectors (*c*_3_^*X*^ and *f*_3_^*X*^, resp.) are constructed as *k*-tuples of all *W*_3_ which are identified in the sequence *X*. The sequence *X* is travelled in a window of size *k* = 3. The word within each window is compared with the words of *W*_3_. In this case, *n* − *k* + 1 = 4 comparisons are necessary (*ATA*, *TAT*, *ATA*, *TAC*):

(4) $$\begin{array}{c} W_{3}=\left\{ATA,TAT,TAC,AAA,\ldots \right\},\\ c_{3}^{X}=\left(2,1,1,0,\ldots \right),\\ f_{3}^{X}=\left(0.5,0.25,0.25,0,\ldots \right). \end{array}$$

### 2.2. The* k-mers* Counting Method

In the* k-mers* counting method, we use the term* k-mer* to represent the words, or *k*-tuples. This method presents a considerably greater speed in relation to conventional methods, which require alignment [10]. Its algorithm, implemented to determine the number of* k-mers* shared by two sequences, is *O*(*n*), for sequences of size *n*. Differently, the conventional algorithms, which require alignment, are *O*(*n* ^2^).

This algorithm uses, in general, a little different alphabet. In most cases, the alphabet used is a variation of the default alphabet. Known by compressed alphabets, these alphabets contain symbols that denote classes that correspond to two or more different types of residues (each residue is represented by a letter).

For amino acids sequences, a compressed alphabet *C* of size *N* is a partition of the default amino acids alphabet *A*, which contains 20 letters, in *N* disjoined classes containing similar amino acids.  Table 1, extracted from [10], shows some examples of compressed alphabets.

With the use of compressed alphabets the identity is highly conserved. Pairs of related sequences have always a greater or equal identity and, therefore, more* k-mers* in common in an alphabet smaller than the default alphabet. An example of this characteristic can be seen in Table 2.

In Table 2, the upper and lower alignment are the same. The difference is that the first uses *A*, as default amino acids alphabet, while the second uses the compressed alphabet SE-V(10), whose members of the classes are represented by their first letters in alphabetical order—*I*, *L*, *M*, and *V* are shown as *I*, for example. The columns which are fully conserved are indicated with capital letters and with an asterisk below. The number of conserved columns (*k* = 1) increased from 12 in *A* to 19 in SE-V(10). For *k* = 3, the number of fully conserved* k-mers* increased from 4 in *A* to 10 in SE-V(10) and, for *k* = 4, from 3 to 8.

The choice of the alphabet and the value of *k* is based on statistics and has strong impact on the number of conserved identities. If the alphabet is selected, which means that there is a high probability of residues replacements of the same class and a low probability of residues replacements from distinct classes, then we probably have an increase in the number of identities detected. Moreover, the value of *k* confines this increasing in regions of continuous identity. As the sequences differ, the number of conserved* k-mers* is reduced, reaching a limit compared to the expected number of no related sequences. The use of compressed alphabets increases the likelihood of this limit be reached at a greater evolutionary distance. Subtle choices of the size of this alphabet and the value of *k* can provide a better measure of similarity.

The following equation shows how we calculate the similarity between sequences *X* and *Y* by the* k-mers* counting method:

(5) $$\begin{array}{c} F\left(X,Y\right)=\frac{\sum_{\tau }\min\left[n_{X}\left(\tau \right),n_{Y}\left(\tau \right)\right]}{\left[\min\left(L_{X},L_{Y}\right)-k+1\right]}. \end{array}$$

Here *τ* is a* k-mer*, *L*_*X*_ and *L*_*Y*_ are the sequences lengths, and *n*_*X*_(*τ*) and *n*_*Y*_(*τ*) are the number of times *τ* appears in *X* and *Y*, respectively.

### 2.3. Multiple Sequence Similarities Calculation

In any application that performs a comparison between multiple sequences, either by multiple alignment or just by the construction of phylogenetic trees, we perform the similarity calculation in many independent sequences. Thus, the *F* value is obtained for all pairs of sequences involved in the processing. As *F*(*X*, *Y*) equals *F*(*Y*, *X*), this value is calculated once, by two nested loops, as

for (i = 1; i < num_seq; ++i)

for (j = 0; j < i; ++i)

M[i,j] = F(i,j)

All obtained values of *F* are, therefore, stored in a triangular matrix *M*.

### 2.4. Parallel Algorithm

In an application, the amount of sequences in comparison can be huge, making its implementation unfeasible in a single machine, even with the use of low computational complexity methods, as the* k-mers* counting method. For this reason, we propose a parallel algorithm that calculates the similarities of multiple pairs of sequences using the* k-mers* counting method.

This algorithm dynamically divides the computation among existing processors through a master-slave approach. This parallelism is performed distributing the similarity calculation of sequence pairs to available slaves. It is possible because each similarity pair calculation is independent of other calculations of similarity pair.

Initially, the master sends all sequences by broadcasting to all slaves. Using broadcast the master can reduce the overhead with the messages exchange, decreasing the communication time between processors.

**Figure 1:**
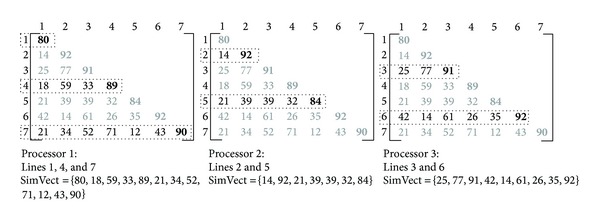
Example of how the similarities matrix calculation is distributed between the slaves.

The tasks distribution is based on the processor identifier, assigning to each slave the calculation of specific lines of the triangular matrix of similarities. The way this matrix is obtained is exemplified in Figure 1. Each slave initially calculates the corresponding line of the identifier, in a *p*-step loop, where *p* is the number of processors. In Figure 1, we have seven sequences and, hence, seven lines in the matrix. The first slave is responsible for the calculation of the lines 1, 4, and 7, the second slave by lines 2 and 5 and the third slave by lines 3 and 6. The gray values are the obtained results in other slaves which will only be joined in the master for the creation of the similarities matrix.

**Figure 2:**
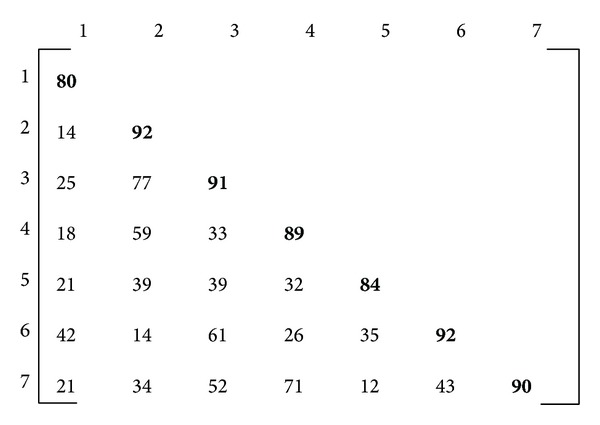
Similarities matrix that is built by the master processor.

During the matrix lines calculation, each slave stores the results of all lines in a single vector (SimVect) which is sent to master. The master receives all slave vectors and, from these vectors and the identifier the slave has sent, it builds the similarities matrix. From the previous example, the obtained similarities matrix is shown in Figure 2.

**Figure 3:**
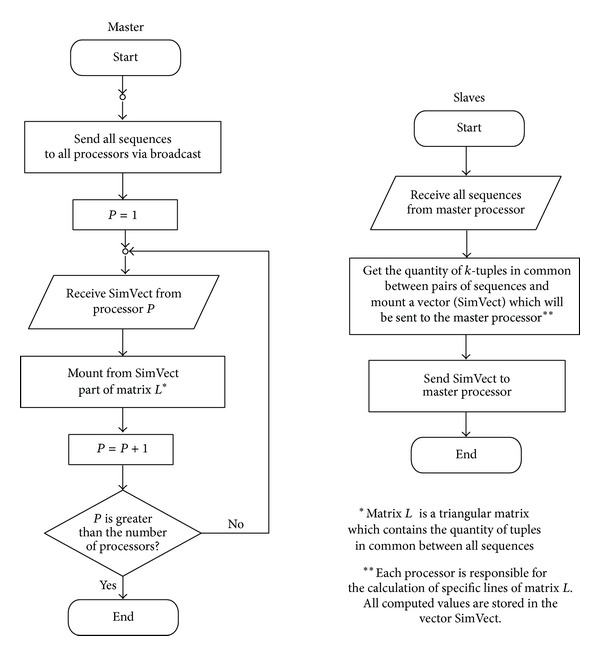
Flowchart of the parallel algorithm for multiple sequence similarities calculation.

In Figure 3 is showed the flowchart of the algorithm which performs the task in Figures 1 and 2.

## 3. Results

### 3.1. Performance Evaluation

Many tests were performed to measure the performance of the proposed algorithms. The datasets used in these experiments have differences only on the number of sequences to be aligned and their lengths. The sequences used were extracted from the NCBI database (http://www.ncbi.nlm.nih.gov/). For each performed test, we describe specific information of the dataset used, as the number of sequences and the average and maximum length of the sequences.

Tests were performed on a Beowulf cluster running under Linux Debian. The Beowulf cluster consists of 15 nodes, each one composed by one AMD Athlon XP 2100+ processor with 1 GB of RAM memory. The nodes are connected with a dedicated 10/100 Fast Ethernet switch. The tests were performed with 1, 2, 4, 8, and 15 nodes. The run times were measured by executing them in stand alone mode, to ensure exclusive use of the communication and processors CPU and memory.

In order to verify the proposed algorithm performance, we executed tests with four different datasets. For each dataset, we verified the algorithm scalability when executed in a crescent machine number.

**Figure 4:**
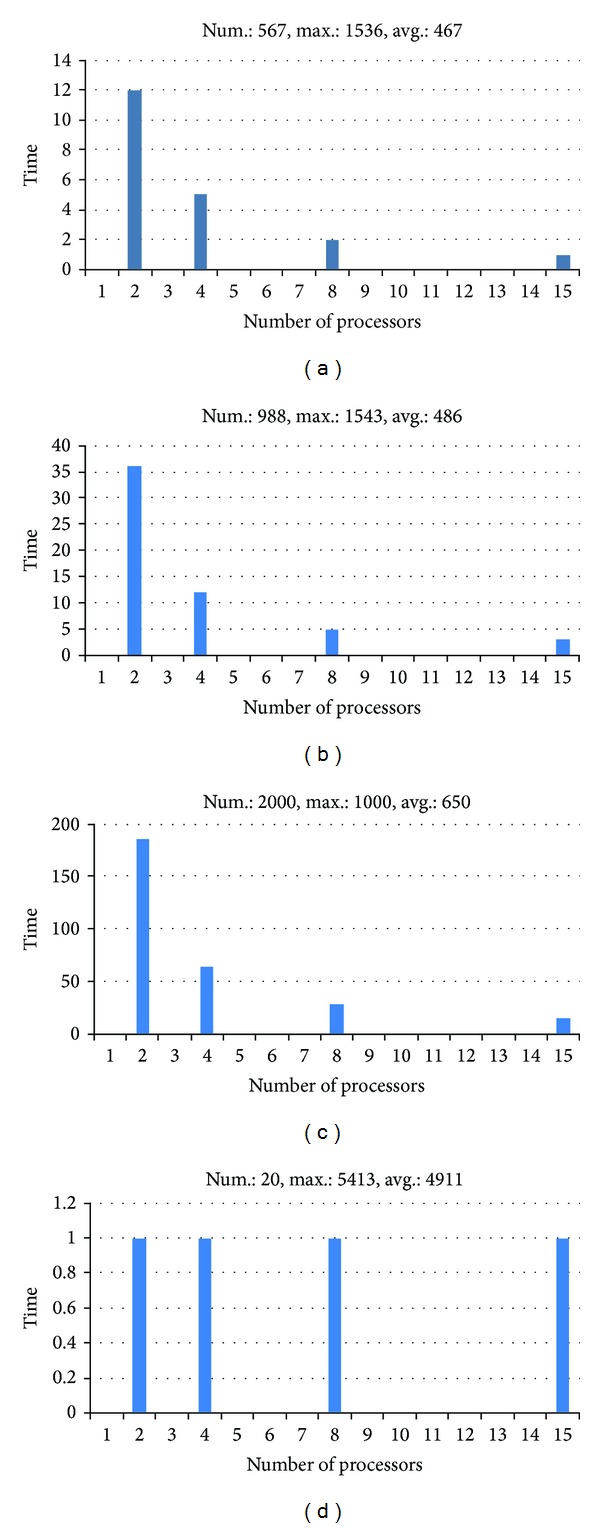
Processing time, in seconds, of the parallel algorithm for 2, 4, 8, and 15 nodes executing with four different datasets.

The first three graphics ((a), (b) and (c)) illustrated in Figure 4 show an almost linear speedup for tests with more than 500 sequences. Notice that the minimum system set for the parallel algorithm execution consists of two nodes, because such algorithm has a master-slave model. One machine (the master) is responsible for data management. The performance difference between the sequential algorithm and the parallel one, when run on a minimum system, is almost zero. For this reason, we only showed the tests performed with the parallel algorithm from the minimum system of two nodes until the maximum number of nodes in the cluster.

The graphic (d), also in Figure 4, illustrates also a constant performance, regardless of the number of machines, for an entry with few sequences. In this case, the sequential implementation of the algorithm is so fast for that input (approximately 1 second) that the speedup achieved with tasks division is close to the time spent with messages exchanges.

Comparing the minimum system (2 nodes) with the maximum system (15 nodes) we have an increase of 14 nodes and, approximately, a 12x speedup. Therefore, it can be noticed that the parallel algorithm presents excellent results with a nearly linear speedup.

## 4. Discussion

In this work, we presented a parallel strategy to calculate similarities between multiple pairs of sequences using the* k-mers* couting method, a low computational complexity method with good biological results. This calculation is used, for example, in multiple sequence alignment tools. The proposed parallel algorithm has been implemented for distributed memory systems, due to the wide use of Beowulf clusters in genomic research laboratories.

The tests performed show that the algorithm presents a good scalability and a nearly linear speedup. With the use of 14 processing nodes (slaves), the system achieved a 12x speedup. This speedup is justified by the total independence of the scheduled tasks and by the good load balancing obtained with the triangular matrix lines distribution.

Additionally, the communication cost is minimized because the computed data in slaves are sent to master in a single message. Considering the 12x speedup achieved and using Amdahl's law [11] we can estimate that about 1.5% of the processes in the system are unparallelized, which reinforces the need of obtained optimization with the concentrated communication, avoiding message overload.

## Figures and Tables

**Table 1:** Examples of compressed alphabets.

Alphabet (*N*)	Classes
SE-B (14)	A, C, D, EQ, FY, G, H, IV, KR, LM, N, P, ST, W
SE-B (10)	AST, C, DN, EQ, FY, G, HW, ILMV, KR, P
SE-V (10)	AST, C, DEN, FY, G, H, ILMV, KQR, P, W
Li-A (10)	AC, DE, FWY, G, HN, IV, KQR, LM, P, ST
Li-B (10)	AST, C, DEQ, FWY, G, HN, IV, KR, LM, P
Solis-D (10)	AM, C, DNS, EKQR, F, GP, HT, IV, LY, W
Solis-G (10)	AEFIKLMQRVW, C, D, G, H, N, P, S, T, Y
Murphy (10)	A, C, DENQ, FWY, G, H, ILMV, KR, P, ST
SE-B (8)	AST, C, DHN, EKQR, FWY, G, ILMV, P
SE-B (6)	AST, CP, DEHKNQR, FWY, G, ILMV
Dayhoff (6)	AGPST, C, DENQ, FWY, HKR, ILMV

**Table 2:** Comparison between the use of the alphabet *A* and the compressed alphabet SE-V (10).

Seq1: sAaNiLvGEnlvcKvaDFGLARl	
Seq2: aArNiLvGEnyicKvaDFGLARl	
Seq3: $\underset{\;}{\text{a}}\underset{*}{\text{A}}\underset{\;}{\text{r}}\underset{*}{\text{N}}\underset{\;}{\text{v}}\underset{*}{\text{L}}\underset{\;}{\text{i}}\underset{*}{\text{G}}\underset{*}{\text{E}}\underset{\;}{\text{d}}\underset{\;}{\text{n}}\underset{\;}{\text{v}}\underset{\;}{\text{a}}\underset{*}{\text{K}}\underset{\;}{\text{i}}\underset{\;}{\text{c}}\underset{*}{\text{D}}\underset{*}{\text{F}}\underset{*}{\text{G}}\underset{*}{\text{L}}\underset{*}{\text{A}}\underset{*}{\text{R}}\underset{\;}{\text{v}}$	
Using the default amino acids alphabet	

Seq1: AAaNILIGENlIcKIaDFGLARI	
Seq2: AArNILIGENyIcKIaDFGLARI	
Seq3: $\underset{*}{\text{A}}\underset{*}{\text{A}}\underset{\;}{\text{r}}\underset{*}{\text{N}}\underset{*}{\text{I}}\underset{*}{\text{L}}\underset{*}{\text{I}}\underset{*}{\text{G}}\underset{*}{\text{E}}\underset{*}{\text{N}}\underset{\;}{\text{n}}\underset{*}{\text{I}}\underset{\;}{\text{a}}\underset{*}{\text{K}}\underset{*}{\text{I}}\underset{\;}{\text{c}}\underset{*}{\text{D}}\underset{*}{\text{F}}\underset{*}{\text{G}}\underset{*}{\text{L}}\underset{*}{\text{A}}\underset{*}{\text{R}}\underset{*}{\text{I}}$	
Using the compressed alphabet SE-V (10)	
(each class member is represented by the first letter in alphabetical order)	
